# Common germline polymorphisms associated with breast cancer-specific survival

**DOI:** 10.1186/s13058-015-0570-7

**Published:** 2015-04-22

**Authors:** Ailith Pirie, Qi Guo, Peter Kraft, Sander Canisius, Diana M Eccles, Nazneen Rahman, Heli Nevanlinna, Constance Chen, Sofia Khan, Jonathan Tyrer, Manjeet K Bolla, Qin Wang, Joe Dennis, Kyriaki Michailidou, Michael Lush, Alison M Dunning, Mitul Shah, Kamila Czene, Hatef Darabi, Mikael Eriksson, Dieter Lambrechts, Caroline Weltens, Karin Leunen, Chantal van Ongeval, Børge G Nordestgaard, Sune F Nielsen, Henrik Flyger, Anja Rudolph, Petra Seibold, Dieter Flesch-Janys, Carl Blomqvist, Kristiina Aittomäki, Rainer Fagerholm, Taru A Muranen, Janet E Olsen, Emily Hallberg, Celine Vachon, Julia A Knight, Gord Glendon, Anna Marie Mulligan, Annegien Broeks, Sten Cornelissen, Christopher A Haiman, Brian E Henderson, Frederick Schumacher, Loic Le Marchand, John L Hopper, Helen Tsimiklis, Carmel Apicella, Melissa C Southey, Simon S Cross, Malcolm WR Reed, Graham G Giles, Roger L Milne, Catriona McLean, Robert Winqvist, Katri Pylkäs, Arja Jukkola-Vuorinen, Mervi Grip, Maartje J Hooning, Antoinette Hollestelle, John WM Martens, Ans MW van den Ouweland, Federick Marme, Andreas Schneeweiss, Rongxi Yang, Barbara Burwinkel, Jonine Figueroa, Stephen J Chanock, Jolanta Lissowska, Elinor J Sawyer, Ian Tomlinson, Michael J Kerin, Nicola Miller, Hermann Brenner, Katja Butterbach, Bernd Holleczek, Vesa Kataja, Veli-Matti Kosma, Jaana M Hartikainen, Jingmei Li, Judith S Brand, Keith Humphreys, Peter Devilee, Robert AEM Tollenaar, Caroline Seynaeve, Paolo Radice, Paolo Peterlongo, Siranoush Manoukian, Filomena Ficarazzi, Matthias W Beckmann, Alexander Hein, Arif B Ekici, Rosemary Balleine, Kelly-Anne Phillips, kConFab Investigators, Javier Benitez, M Pilar Zamora, Jose Ignacio Arias Perez, Primitiva Menéndez, Anna Jakubowska, Jan Lubinski, Jacek Gronwald, Katarzyna Durda, Ute Hamann, Maria Kabisch, Hans Ulrich Ulmer, Thomas Rüdiger, Sara Margolin, Vessela Kristensen, Siljie Nord, NBCS Investigators, D Gareth Evans, Jean Abraham, Helena Earl, Christopher J Poole, Louise Hiller, Janet A Dunn, Sarah Bowden, Rose Yang, Daniele Campa, W Ryan Diver, Susan M Gapstur, Mia M Gaudet, Susan Hankinson, Robert N Hoover, Anika Hüsing, Rudolf Kaaks, Mitchell J Machiela, Walter Willett, Myrto Barrdahl, Federico Canzian, Suet-Feung Chin, Carlos Caldas, David J Hunter, Sara Lindstrom, Montserrat Garcia-Closas, Fergus J Couch, Georgia Chenevix-Trench, Arto Mannermaa, Irene L Andrulis, Per Hall, Jenny Chang-Claude, Douglas F Easton, Stig E Bojesen, Angela Cox, Peter A Fasching, Paul DP Pharoah, Marjanka K Schmidt

**Affiliations:** Centre for Cancer Genetic Epidemiology, Department of Public Health and Primary Care, University of Cambridge, 2 Wort’s Causeway, Cambridge, CB1 8RN UK; Centre for Cancer Genetic Epidemiology, Department of Oncology, University of Cambridge, 2 Wort’s Causeway, Cambridge, CB1 8RN UK; Program in Genetic Epidemiology and Statistical Genetics, Department of Epidemiology, Harvard School of Public Health, 677 Huntington Avenue, Boston, MA 02115 USA; Department of Epidemiology, Harvard School of Public Health, 677 Huntington Avenue, Boston, MA 02115 USA; Netherlands Cancer Institute, Antoni Van Leeuwenhoek Hospital, Plesmanlaan 121, 1066 CX Amsterdam, The Netherlands; Faculty of Medicine, University of Southampton, Highfield Campus, Southampton, SO17 1BJ UK; Division of Genetics and Epidemiology, Institute of Cancer Research, 15 Cotswold Road, Sutton, SM2 5NG Surrey, UK; Department of Obstetrics and Gynecology, University of Helsinki and Helsinki University Central Hospital, Haartmaninkatu 8, FIN-00029 HUS Helsinki, Finland; Department of Medical Epidemiology and Biostatistics, Karolinska Institutet, Nobels väg 12A, Stockholm, 17177 Sweden; Vesalius Research Center (VRC), Vib, Herestraat 49, 3000 Leuven, Belgium; Laboratory for Translational Genetics, Department of Oncology, University of Leuven, Herestraat 49, 3000 Leuven, Belgium; Oncology Department, University Hospital Gasthuisberg, Herestraat 49, 3000 Leuven, Belgium; Copenhagen General Population Study, Herlev Hospital, Copenhagen University Hospital, Herlev Ringvej 75, DK-2730 Herlev, Copenhagen Denmark; Department of Clinical Biochemistry, Herlev Hospital, Copenhagen University Hospital, Herlev Ringvej 75, DK-2730 Herlev, Copenhagen Denmark; Faculty of Health and Medical Sciences, University of Copenhagen, Blegdamsvej 3, DK-2220 Copenhagen, Denmark; Department of Breast Surgery, Herlev Hospital, Copenhagen University Hospital, Herlev Ringvej 75, DK-2730 Herlev, Copenhagen Denmark; Division of Cancer Epidemiology, German Cancer Research Center (DKFZ), Im Neuenheimer Feld 280, 69120 Heidelberg, Germany; Department of Cancer Epidemiology/Clinical Cancer Registry and Institute for Medical Biometrics and Epidemiology, University Clinic Hamburg-Eppendorf, Martinistrasse 52, 20246 Hamburg, Germany; Department of Oncology, Helsinki University Central Hospital, Sairaalatie 8, 08 200 LOHJA Helsinki, Finland; Department of Clinical Genetics, Helsinki University Central Hospital, Sairaalatie 8, 08 200 LOHJA Helsinki, Finland; Department of Health Sciences Research, Mayo Clinic, 200 First Street SW, Rochester, MN 55905 USA; Department of Laboratory Medicine and Pathology, Mayo Clinic, 200 First Street SW, Rochester, MN 55905 USA; Prosserman Centre for Health Research, Lunenfeld-Tanenbaum Research Institute, Mount Sinai Hospital, 600 University Avenue, Toronto, ON M5G 1X5 Canada; Division of Epidemiology, Dalla Lana School of Public Health, University of Toronto, 155 College Street, Toronto, ON M5T 3M7 Canada; Ontario Cancer Genetics Network, Lunenfeld-Tanenbaum Research Institute, Mount Sinai Hospital, 600 University Avenue, Toronto, ON M5G 1X5 Canada; Department of Laboratory Medicine and Pathobiology, University of Toronto, 1 King’s College Circle, Toronto, ON M5S 1A8 Canada; Laboratory Medicine Program, University Health Network, 200 Elizabeth Street, Toronto, ON M5G 2C4 Canada; Department of Preventive Medicine, Keck School of Medicine, University of Southern California, 1975 Zonal Avenue, Los Angeles, CA 90033 USA; Cancer Research Center of Hawaii, University of Hawaii, 701 Ilalo Street, Honolulu, HI 96813 USA; Centre for Molecular, Environmental, Genetic and Analytic Epidemiology, Melbourne School of Population Health, The University of Melbourne, 207 Bouverie Street, Melbourne, VIC 3010 Australia; Department of Pathology, The University of Melbourne, 207 Bouverie Street, Melbourne, VIC 3010 Australia; Academic Unit of Pathology, Department of Neuroscience, University of Sheffield, 385a Glossop Road, Sheffield, S10 2HQ UK; CRUK/YCR Sheffield Cancer Research Centre, Department of Oncology, University of Sheffield, Beech Hill Road, Sheffield, S10 2RX UK; Cancer Epidemiology Centre, The Cancer Council Victoria, 615 St Kilda Road, Melbourne, VIC 3004 Australia; Anatomical Pathology, The Alfred Hospital, Commercial Road, Melbourne, VIC 3007 Australia; Laboratory of Cancer Genetics and Tumor Biology, Department of Clinical Genetics and Biocenter Oulu, University of Oulu, Oulu University Hospital, Kajaanintie 50, FI-90220 Oulu, Finland; Department of Oncology, Oulu University Hospital, University of Oulu, Kajaanintie 50, FI-90220 Oulu, Finland; Department of Surgery, Oulu University Hospital, University of Oulu, Kajaanintie 50, FI-90220 Oulu, Finland; Department of Medical Oncology, Family Cancer Clinic, Erasmus McCancer Institute, ’s-Gravendijkwal 230, 3015 CE Rotterdam, The Netherlands; Department of Obstetrics and Gynecology, University of Heidelberg, Voßstrasse 9, 69115 Heidelberg, Germany; National Center for Tumor Diseases, University of Heidelberg, Im Neuenheimer Feld 460, 69120 Heidelberg, Germany; Molecular Epidemiology Group, German Cancer Research Center (DKFZ), Im Neuenheimer Feld 280, 69120 Heidelberg, Germany; Division of Cancer Epidemiology and Genetics, National Cancer Institute, 9000 Rockville Pike, Bethesda, MD 20892 USA; Core Genotyping Facility, Frederick National Laboratory for Cancer Research, 8717 Grovemont Circle, Gaithersburg, MD 20877 USA; Department of Cancer Epidemiology and Prevention, M. Sklodowska-Curie Memorial Cancer Center and Institute of Oncology, Roentena 5, 02-781 Warsaw, Poland; Division of Cancer Studies, NIHR Comprehensive Biomedical Research Centre, Guy’s and St. Thomas’ NHS Foundation Trust in Partnership with King’s College London, Guy’s Campus, SE1 1UL London, UK; Wellcome Trust Centre for Human Genetics and Oxford Biomedical Research Centre, University of Oxford, Roosevelt Drive, Oxford, OX3 7BN UK; Clinical Science Institute, University Hospital Galway, Newcastle Road, Galway, Ireland; Division of Clinical Epidemiology and Aging Research, German Cancer Research Center (DKFZ), Im Neuenheimer Feld 581, 69120 Heidelberg, Germany; German Cancer Consortium (DKTK), Im Neuenheimer Feld 280, 69120 Heidelberg, Germany; Saarland Cancer Registry, Präsident Baltz Strasse 5, 66119 Saarbrücken, Germany; School of Medicine, Institute of Clinical Medicine, Oncology and Cancer Center, Kuopio University Hospital, Puijonlaaksontie 2, 70210 Kuopio, Finland; School of Medicine, Institute of Clinical Medicine, Pathology and Forensic Medicine and Cancer Center of Eastern Finland, University of Eastern Finland, Yliopistonranta 1C, 70210 Kuopio, Finland; Imaging Center, Department of Clinical Pathology, Kuopio University Hospital, Puijonlaaksontie 2, 70210 Kuopio, Finland; Department of Human Genetics and Department of Pathology, Leiden University Medical Center, Albinusdreef 2, 2333 ZA Leiden, The Netherlands; Department of Surgical Oncology, Leiden University Medical Center, Albinusdreef 2, 2333 ZA Leiden, The Netherlands; Unit of Molecular Bases of Genetic Risk and Genetic Testing, Department of Preventive and Predictive Medicine, Fondazione IRCCS Istituto Nazionale Dei Tumori (INT), Via Adamello 16, Milan, 20139 Italy; IFOM, Fondazione Istituto FIRC Di Oncologia Molecolare, Via Adamello 16, 20139 Milan, Italy; Unit of Medical Genetics, Department of Preventive and Predictive Medicine, Fondazione IRCCS Istituto Nazionale Dei Tumori (INT), Via Adamello 16, Milan, 20139 Italy; Cogentech Cancer Genetic Test Laboratory, Via Adamello 16, 20139 Milan, Italy; Department of Gynecology and Obstetrics, University Hospital Erlangen, Friedrich-Alexander University Erlangen-Nuremberg, Comprehensive Cancer Center Erlangen-Emn, Universitaetsstrasse 21-23, 91054 Erlangen, Germany; Institute of Human Genetics; University Hospital Erlangen, Friedrich-Alexander University Erlangen-Nuremberg, Comprehensive Cancer Center Erlangen-Emn, Universitaetsstrasse 21-23, 91054 Erlangen, Germany; Western Sydney and Nepean Blue Mountains Local Health Districts, Westmead Millennium Institute for Medical Research, University of Sydney, 176 Hawkesbury Road, Sydney, NSW 2145 Australia; Peter Maccallum Cancer Center, 2 St Andrews Place, Melbourne, VIC 3002 Australia; Sir Peter Maccallum Department of Oncology, University of Melbourne, 2 St Andrews Place, Melbourne, VIC 3002 Australia; Human Genetics Group, Human Cancer Genetics Program, Spanish National Cancer Research Centre (CNIO), Calle de Melchor Fernández, Almagro, 3, 28029 Madrid Spain; Centro de Investigación En Red De Enfermedades Raras (CIBERER), Calle de Álvaro de Bazán, 10 Bajo, 46010 Valencia, Spain; Servicio de Oncología Médica, Hospital Universitario La Paz, Paseo de la Castellana, 261, 28046 Madrid, Spain; Servicio de Cirugía General y Especialidades, Hospital Monte Naranco, Avenida Doctores Fernández Vega, 107, 33012 Oviedo, Spain; Servicio de Anatomía Patológica, Hospital Monte Naranco, Avenida Doctores Fernández Vega, 107, 33012 Oviedo, Spain; Department of Genetics and Pathology, Pomeranian Medical University, ul. Rybacka 1, Szczecin, Poland; Molecular Genetics of Breast Cancer, German Cancer Research Center (DKFZ), Im Neuenheimer Feld 280, 69120 Heidelberg, Germany; Frauenklinik der Stadtklinik Baden-Baden, Balger Strasse 50, 76532 Baden-Baden, Germany; Institute of Pathology, Städtisches Klinikum Karlsruhe, Moltkestrasse 90, 76133 Karlsruhe, Germany; Department of Oncology - Pathology, Karolinska Institutet, Tomtebodavägen 23b, Stockholm, 171 65 Sweden; Faculty of Medicine (Faculty Division Ahus), University of Oslo (UiO), Problemveien 7, Oslo, 0313 Norway; Department of Genetics, Institute for Cancer Research, Oslo University Hospital, Radiumhospitalet, Montebello, 0379 Oslo Norway; Genomic Medicine, Manchester Academic Health Science Centre, University of Manchester, Central Manchester Foundation Trust, St. Mary’s Hospital, Oxford Road, Manchester, M13 9WL UK; Cambridge Experimental Cancer Medicine Centre, Robinson Way, Cambridge, CB2 0RE UK; Cambridge Breast Unit and NIHR Cambridge Biomedical Research Centre, University of Cambridge NHS Foundation Hospitals, Hills Road, Cambridge, CB2 0QQ UK; Warwick Clinical Trials Unit, University of Warwick, Gibbet Hill Campus, Coventry, CV4 7AL UK; Cancer Research UK Clinical Trials Unit, Institute for Cancer Studies, the University of Birmingham, Vincent Drive, Edgbaston, Birmingham, B15 2TT UK; Early Detection Research Group, Division of Cancer Prevention National Cancer Institute, 9609 Medical Center Drive, Bethesda, MD 20892 USA; Department of Biology, University of Pisa, Lungarno Pacinotti 43, 56126 Pisa, Italy; Epidemiology Research Program, American Cancer Society, 250 Williams Street, Atlanta, GA 30303 USA; Division of Biostatistics and Epidemiology, University of Massachusetts-Amherst School of Public Health and Health Sciences, 715 N Pleasant Street, Amherst, MA 01002 USA; Channing Division of Network Medicine, Department of Medicine, Brigham and Women’s Hospital, 75 Francis Street, Boston, MA 02115 USA; Department of Nutrition, Harvard School of Public Health, 655 Huntington Avenue, Boston, MA 02115 USA; Genomic Epidemiology Group, German Cancer Research Center (DKFZ), Im Neuenheimer Feld 280, 69120 Heidelberg, Germany; Breast Cancer Functional Genomics Laboratory, Cancer Research UK Cambridge Institute, University of Cambridge, Li Ka Shing Centre, Robinson Way, CB2 0RE UK; Program in Molecular and Genetic Epidemiology, Harvard School of Public Health, 677 Huntington Avenue, Boston, MA 02115 USA; Breakthrough Breast Cancer Research Centre, Division of Breast Cancer Research, The Institute of Cancer Research, 123 Old Brompton Road, London, SW7 3RP UK; Department of Genetics, Qimr Berghofer Medical Research Institute, 300 Herston Road, Brisbane, QLD 4006 Australia; Department of Molecular Genetics, University of Toronto, 1 King’s College Circle, Toronto, ON M5S 1A8 Canada; David Geffen School of Medicine, Department of Medicine, Division of Hematology and Oncology, University of California at Los Angeles, 10833 Le Conte Avenue, Los Angeles, CA 90095 USA

## Abstract

**Introduction:**

Previous studies have identified common germline variants nominally associated with breast cancer survival. These associations have not been widely replicated in further studies. The purpose of this study was to evaluate the association of previously reported SNPs with breast cancer-specific survival using data from a pooled analysis of eight breast cancer survival genome-wide association studies (GWAS) from the Breast Cancer Association Consortium.

**Methods:**

A literature review was conducted of all previously published associations between common germline variants and three survival outcomes: breast cancer-specific survival, overall survival and disease-free survival. All associations that reached the nominal significance level of *P* value <0.05 were included. Single nucleotide polymorphisms that had been previously reported as nominally associated with at least one survival outcome were evaluated in the pooled analysis of over 37,000 breast cancer cases for association with breast cancer-specific survival. Previous associations were evaluated using a one-sided test based on the reported direction of effect.

**Results:**

Fifty-six variants from 45 previous publications were evaluated in the meta-analysis. Fifty-four of these were evaluated in the full set of 37,954 breast cancer cases with 2,900 events and the two additional variants were evaluated in a reduced sample size of 30,000 samples in order to ensure independence from the previously published studies. Five variants reached nominal significance (*P* <0.05) in the pooled GWAS data compared to 2.8 expected under the null hypothesis. Seven additional variants were associated (*P* <0.05) with ER-positive disease.

**Conclusions:**

Although no variants reached genome-wide significance (*P* <5 x 10^−8^), these results suggest that there is some evidence of association between candidate common germline variants and breast cancer prognosis. Larger studies from multinational collaborations are necessary to increase the power to detect associations, between common variants and prognosis, at more stringent significance levels.

**Electronic supplementary material:**

The online version of this article (doi:10.1186/s13058-015-0570-7) contains supplementary material, which is available to authorized users.

## Introduction

Breast cancer is the most commonly diagnosed cancer in women, in the world, with an estimated 1.67 million new cancer cases diagnosed in 2012. Breast cancer mortality is the second most common cancer-related death in women in the more developed regions of the world and accounts for 15.4% of cancer-related deaths in women [1]. Breast cancer outcome is affected by several factors including: age, tumour size, tumour grade, extent of local and distal spread at diagnosis, oestrogen receptor (ER) status, human epidermal growth factor receptor 2 (HER2) status and treatment received. It is also likely that inherited host characteristics, such as genetic variants, are important [2].

The association between common germline genetic variation and breast cancer survival has been examined in many candidate gene studies investigating genes in pathways known to be involved in breast cancer [3]. These studies have identified numerous single nucleotide polymorphisms (SNPs) associated with outcome at nominal significance levels, but none have been widely replicated in further studies. The exceptions to this are three genome-wide association studies (GWAS) [4-6] and a study from the Breast Cancer Association Consortium, which had substantial power to detect associated variants with large effect sizes (hazard ratio (HR) >2) [7]. Two of those GWAS have reported significant associations for three polymorphisms (rs9934948, rs3784099, rs4778137) [4,6]. The aim of this study was to evaluate the association of previously reported SNPs with prognosis using data from a hypothesis-generating pooled analysis of eight breast cancer survival GWAS from ten studies including 37,954 breast cancer cases [8].

## Methods

### Literature review

Studies reporting common polymorphisms associated with breast cancer prognosis were identified by searching both Google Scholar and Pubmed. We searched Google Scholar using the search terms: ‘breast cancer’, ‘survival’, ‘prognosis’, ‘polymorphisms’ and ‘SNPs’. The search terms for Pubmed were ‘breast cancer’ AND (‘survival’ OR ‘prognosis’) AND (‘polymorphism’ OR ‘SNP’). The references of all identified studies were then individually interrogated for any additional studies. The search was last updated on 6 June 2014. We considered studies to be eligible for inclusion if they reported an association between a germline genetic variant and at least one of the following end points: overall survival, disease-free survival and breast cancer-specific survival (BCSS). Studies evaluating the prognostic importance of rare high-penetrance variants with minor allele frequency <2% in *BRCA1*, *BRCA2* and *CHEK2* were omitted from the review. Only one study conducted ER subtype-specific analyses.

For the purposes of comparison, all studies that used genetic models that grouped together two genotypes into a single category were defined as using ‘dominance models’. This category includes both dominant and recessive models as each study's definition of a dominant or recessive model is dependent on which allele is the major or minor allele, whether they consider the effect allele to be bi-directional, or whether they focus on only the risk allele.

### Genome-wide association studies

We used data from a combined analysis of eight breast cancer GWAS, from ten studies [9-19], that had genotype data from a genome-wide SNP array and had collected follow-up time data for the 37,954 breast cancer cases [8]. Genotype and sample quality control were carried out separately for each study. In short, SNPs were excluded based on: low call rate, minor allele frequency <1% and significant deviation of genotype frequencies from the Hardy-Weinberg equilibrium. Samples were excluded for: low call rate, ambiguous gender, relatedness and extreme heterozygosity. We also excluded subjects of less than 90% European ancestry. Sample ancestry was determined separately for each GWAS included in the meta-analysis using either principal component analysis, multi-dimensional scaling or LAMP based on ethnicities from HapMap samples. Samples with less than 90% European ancestry were excluded. As different genotyping arrays had been used for the different studies, imputation had been performed using a reference panel from the 1000 Genomes Project [8,20]. We utilised the imputed data for the SNPs of interest in this study. Details of the pooled studies are shown in Additional files 1 and 2.

Cox proportional hazards models were fitted to assess the association of genotype with breast cancer-specific mortality under a co-dominant (log-additive) genetic model using the likelihood ratio test. The models were adjusted for principal components in order to minimise the effect of population substructure, and the Collaborative Oncological Gene-environment Study (COGS) [16] dataset was stratified by study. Each survival GWAS was analysed separately and the results were harmonised and combined using a standard inverse-variance weighted fixed-effects meta-analysis. In order to compare the results with the published associations we used a one-sided test based on the reported direction of effect. In the initial analysis all 56 SNPs' models were unadjusted for prognostic factors. However, we conducted multivariable analysis of the previously reported SNPs that were significantly associated with survival adjusting for age, stage and grade using 29,360 samples from the COGS study.

## Results

### Literature review

We identified 46 publications reporting nominally significant associations between 62 germline variants and survival after a breast cancer diagnosis. Details of each variant and the reported association with breast cancer prognosis are shown in Additional file 3. The median sample size was 890 cases; the smallest study had 85 cases and the largest 25,853. Fifty-nine variants were from 44 candidate gene studies and three variants were identified through GWAS. The candidate genes were involved in the following pathways: DNA repair, cell cycle control, matrix metalloproteinases, immune response, drug response, tumour progression, vitamin D receptors and miscellaneous other pathways (Table 1). Findings from the identified publications were infrequently replicated; only six variants out of the 62 were reported in at least one subsequent publication.

**Table 1:** **Previously identified breast cancer survival genes in cancer-related pathways**

**Pathway**	**Nearest gene**	**References**
DNA repair	*XRCC1, XRCC2, XRCC3, RAD51B, LIG4, ERCC2*	[6,28-32]
Cell cycle control	*CCND1, CCND3, PRKAG2, TP53, SIPA1, FGFR2, PPP2R2B*	[28,33-38]
Matrix metalloproteinases	*MMP7, MMP8, MMP2, SERPINE1, TIMP-3*	[23,40-44]
Immune and drug response, metabolism	*Il-10, IL-6, IL-21, MPO, GSTP1, COMT, CYP19A1, CYP1A1, SULT1E1, NEF2L2, TLR4, SLC28A3, CD24, CD44, NQO1*	[14,22,24,45-57]
Tumour progression	*NOS3, VEGF, NME1, SELE, GNAS1, ZFP36, TGF*	[58-64]
Vitamin D receptors	*RXRA, VDR*	[65,66]
Miscellaneous	*TOX3, MTHFR, COX11, OCA2, PLAUR.*	[4,7,34,65,67]

NB: the genes mentioned here are the candidate genes listed in the previous publications or are the nearest gene to the single nucleotide polymorphism (SNP) and are not necessarily the genes on which the SNPs have a functional effect.

### Meta-analysis findings

Results from the GWAS meta-analysis included 58 of the 62 previously identified variants discussed above. The SNP (rs2886162) was replaced by a perfectly correlated tagSNP (rs2364725, r^2^ = 1). Associations for four of the variants identified: rs4778137 in *OCA2*, rs3803662 in *TOX3*, rs1042522 in *TP53* and rs2479717 in *CCND1* were discovered in studies carried out by the Breast Cancer Association Consortium using sets of samples included in our GWAS meta-analysis. Therefore, we are unable to replicate these associations independently in the full dataset. The substantial sample overlap between the studies that identified associations with rs4778137 and rs3803662 means that there is little to be gained by attempting to replicate their associations in the additional samples included in the meta-analysis. However, the sample sizes in the studies identifying rs1042522 and rs2479717 were relatively small, so we evaluated their association with BCSS in the GWAS meta-analysis omitting the samples from studies used in the original publications. The two SNPs were evaluated in 29,224 and 31,434 samples respectively.

**Figure 1:**
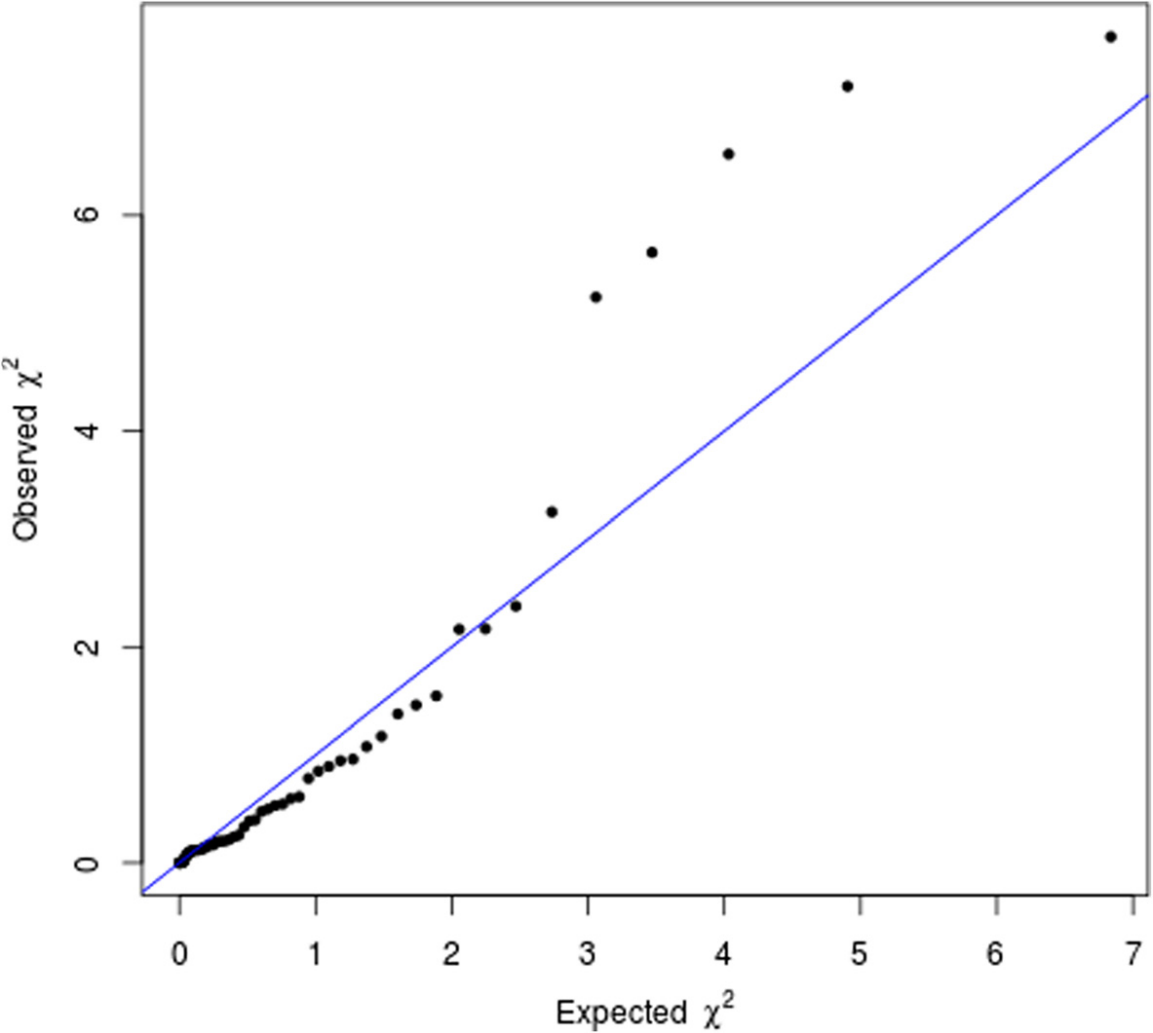
Quantile-quantile plot of results from look-up of previously reported associations in genome-wide association studies. Tests were one-sided with direction assumed from previous association.

The results for the 56 SNPs evaluated in the meta-analysis are presented in Additional file 4. In the analysis of all cases, five SNPs (rs2981582, rs1800566, rs9934948, rs1800470 and rs3775775) were significant with one-sided *P* value <0.05, 51 SNPs were not significant at this nominal *P* value. The most significant association was for rs2981582 in *FGFR2* (per G allele HR 1.09, 90% confidence interval (CI) 1.04 to 1.14, one-sided *P* value = 0.00085). All significantly associated SNPs had good imputation quality (r^2^ = 0.9 to 1). The imputation r^2^ for all 56 SNPs can be found in Additional file 4. No single SNP reached the stringent level of significance generally regarded as genome-wide significant (*P* value <5x10^−8^) but the number of moderately significant associations (5) was somewhat greater than that expected by chance (2.8). This is illustrated by the quantile-quantile plot shown in Figure 1. Seven SNPs not significantly associated with prognosis in all patients were significant in ER-positive disease. We found evidence of ER-positive specific associations with prognosis for seven out of the twelve SNPs nominally associated (*P* <0.05) with survival. These SNPs were not previously identified in patients with specifically ER-positive disease; however, our observations may agree with the previously reported results as most breast cancers are ER positive. We measured the level of heterogeneity between the studies included in the pooled analysis for the 12 SNPs associated with survival. There was moderate evidence of heterogeneity for the SNP rs2981582 (I^2^ = 41.1%, *P* value = 0.084). For all other SNPs there was low heterogeneity (I^2^ < 25%, *P* value >0.2). Details of the SNPs nominally associated with BCSS are shown in Table 2. The results for the nominally associated SNPs adjusted for age, stage and grade are shown in Additional file 5. The HRs for some of the SNPs were attenuated after adjustment. Also, the associations with BCSS of SNPs rs3775775 and rs2333227 were stronger in the multivariable analysis.

**Table 2:** **Previously reported associations replicated in the meta-analysis**

						**All cases**		**ER-negative cases**		**ER-positive cases**	
**SNP**	**Gene**	**Published**	**Model**	**Effect allele**	**Effect allele freq**	**HR (90% CI)**	**One-sided**	**HR (90% CI)**	**One-sided**	**HR (90% CI)**	**One-sided**
							***P*** **value**		***P*** **value**		***P*** **value**
rs2981582	*FGFR2*	Bayraktar *et al.* [34]	Dominance	G	0.57	1.09 (**1.04**-1.14)	**0.00085**	1.08 (**1.00**-1.16)	0.052	1.04 (**0.98**-1.10)	0.15
rs1800566	*NQO1*	Fagerholm *et al*. [14]	Dominance	A	0.19	1.10 (**1.03**-1.17)	**0.0046**	1.14 (**1.03**-1.25)	**0.015**	1.04 (**0.95**-1.13)	0.23
rs9934948	*LOC100506172*	Shu *et al.* [6] (GWAS)	Co-dominance	T	0.15	0.92 (0.86-**0.98**)	**0.011**	0.90 (0.79-**1.01**)	0.059	0.95 (0.86-**1.04**)	0.18
rs1800470	*TGF*	Shu *et al.* [6]	Co-dominance	A	0.61	0.95 (0.91-**0.99**)	**0.030**	0.96 (0.88-**1.04**)	0.20	0.95 (0.88-**1.02**)	0.12
rs3775775	*SULT1E1*	Choi *et al*. [47]	Dominance	G	0.09	1.08 (**1.00**-1.16)	**0.046**	1.17 (**1.03**-1.31)	**0.02**	1.06 (**0.95**-1.17)	0.18
rs700519	*CYP19A1*	Long *et al*. [55]	Dominance	A	0.03	1.10 (**0.98**-1.22)	0.093	1.03 (**0.83**-1.23)	0.40	1.30 (**1.10**-1.50)	**0.0050**
rs731236	*VDR*	Perna *et al*. [66]	Co-dominance	G	0.39	1.04 (**1.00**-1.08)	0.056	1.03 (**0.95**-1.11)	0.28	1.09 (**1.02**-1.16)	**0.017**
rs12900137	CYP19A1	Long *et al*. [55]	Dominance	C	0.05	1.01 (**0.91**-1.11)	0.47	0.94 (**0.78**-1.10)	0.70	1.18 (**1.02**-1.34)	**0.032**
rs10477313	PPP2R2B	Jamshidi *et al.* [35]	Dominance	T	0.12	0.94 (0.87- **1.01**)	0.08	0.92 (0.79-**1.05**)	0.15	0.88 (0.77-**0.99**)	**0.035**
rs2333227	MPO	Ambrosone *et al*. [45]	Dominance	T	0.21	1.03 (**0.97**-1.09)	0.20	0.95 (**0.87**-1.03)	0.78	1.09 (**1.01**-1.17)	**0.036**
rs1902586	CYP19A1	Long *et al*. [55]	Dominance	A	0.05	1.01 (**0.91**-1.11)	0.44	0.99 (**0.83**-1.15)	0.54	1.16 (**1.01**-1.31)	**0.041**
rs28566535	CYP19A1	Long *et al*. [55]	Dominance	C	0.05	1.00 (**0.90**-1.10)	0.51	0.97 (**0.81**-1.13)	0.60	1.15 (**1.00**-1.30)	**0.046**

Hazard ratios are for breast cancer-specific survival using a Cox proportional hazards model corrected for principal components; hazard ratios, confidence intervals and *P* values are from a co-dominant model; *P* values refer to a one-sided test of association in the direction indicated in bold in the 90% CI of the HR; *P* values in bold indicate results that are nominally significant (*P* <0.05). HR, hazard ratio; CI, confidence interval; GWAS, genome-wide association study.

## Discussion

**Figure 2:**
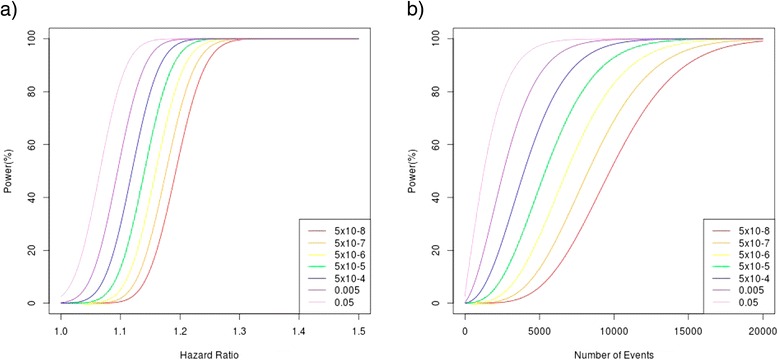
Power (%) to detect true associations with survival time across a range of minor allele frequencies and numbers of events. **(a)** Power (%) to detect true associations with survival time over a range of effect sizes at increasing orders of significance given a minor allele frequency of 0.3 and 2,900 events. We used an imputation r^2^ = 0.8 to account for suboptimal imputation. **(b)** Power (%) to detect true associations with survival time for increasing numbers of events, at increasing orders of significance, given a minor allele frequency of 0.3 and an effect size of 1.1. We used an imputation r^2^ = 0.8 to account for suboptimal imputation.

There have been few studies focused on the replication of sub-genome-wide significant associations identified previously. Previous replication studies have focused on reporting the SNPs with the strongest evidence of association. We have found some evidence to support previously reported associations between common germline genetic variants and breast cancer prognosis. However, the moderate evidence for some variants provides a rationale for continued research efforts to identify such variants. Significant variants were for the most part candidates in cancer-related genes as is shown in Table 1. Despite the larger sample size and therefore increased power to detect true associations with prognosis in comparison to previous studies, a possible reason for associations failing to reach genome-wide significance may still be limited power. Figure 2a illustrates that for our analysis with 2,900 survival events from 37,954 cases, there is limited power to detect associations at stringent significance levels for modest effect sizes based on a variant with a 0.3 minor allele frequency. Figure 2b shows that almost five times as many events would be needed to detect with 80 per cent power at *P* value <10^−8^ an allele with a minor allele frequency of 0.3 that confers a HR of 1.1.

In a two-sided test, five of the previously reported associations with prognosis were significantly associated with BCSS in the GWAS meta-analysis but had discordant directions of effect to the original results. These discrepancies may be caused by differing ethnicity between the sample populations [21] as the meta-analysis is specific to patients with European ancestry whereas the five original studies consider non-European populations [6,22-24]. On the other hand, they may also represent false positive associations in both discovery and replication data.

Many previously published studies used a dominance model to evaluate associations. We only used a co-dominant model to detect association in the GWAS. This is justified because thousands of common variants [25] associated with a range of diseases have been identified using a co-dominant model with little or no evidence for dominance. It seems unlikely that breast cancer survival would differ substantially from other phenotypes in any true, underlying genetic model. Where the true underlying model is co-dominant this approach will maximise statistical power. While it is possible that some variants may be truly associated under a dominance model, for example through loss of heterozygosity of the specific germline variant in the tumour, we would still have reasonable power to detect such an association with the large sample size of the GWAS under a co-dominant model.

A further way to increase power to detect robust associations with prognosis is to reduce the level of heterogeneity in the phenotype. Studies focusing on identifying subtype-specific associations will have increased power to detect variants associated with a particular subtype than an analysis on all patients will have. In particular, studies considering disease subtypes, for example ER-negative disease, may provide valuable information into the reasons for known prognostic differences between subtypes. We identified seven SNPs associated with ER-positive disease. These SNPs were not previously identified in specifically ER-positive disease, however, our observations may agree with the previously reported results as most breast cancers are ER positive. In addition, studies looking at interactions with specific treatments, most notably adjuvant chemotherapy, hormonal therapy and adjuvant radiotherapy, may further inform targeted treatment of subgroups of patients according to their inherited genetic information. Some of the previously reported associations with prognosis were found in specific subgroups of patients; however, as yet the sizes of these studies are limited. Large subtype-specific studies are needed in order to investigate interactions with particular subgroups effectively. The generation of sufficiently large studies to deliver strongly significant results, as well as having good outcome and treatment data to enable powerful subtype-specific analyses, will only be possible by combining data resources through large-scale global collaborations. Case-control studies including approximately 100,000 cases are now being conducted to identify common variants associated with risk. It seems a realistic goal to carry out case-cohort studies of a similar size. Reliable identification of SNPs associated with breast cancer prognosis may help to understand the molecular mechanisms of tumour progression and metastasis. Ultimately, this may lead to the development of new therapeutic targets. Polygenic risk scores based on multiple risk alleles have been shown to have potentially useful discrimination [26]. Similar polygenic prognostic scores may improve discrimination of prognostic and treatment benefit tools such as PREDICT [27].

## Conclusions

We have found limited evidence to support the assertion that germline genetic variation influences outcome after a diagnosis of breast cancer. Large studies with detailed clinical and follow-up information are needed in order to achieve sufficient statistical power to detect associations at stringent significance thresholds. In addition, power can also be increased by reducing the level of phenotype heterogeneity, which will also provide valuable insights into prognostic differences between subgroups.

## Additional files

Additional file 1:
**Study information for GWAS included in meta-analysis** **[** 8 **].**
Additional file 2
**Samples included in meta-analysis by study** **[** 8 **].**
Additional file 3:
**Previously reported associations with breast cancer survival.**
Additional file 4:
**Look-up of previously reported associations in meta-analysis.**
Additional file 5:
**Multivariable analysis results adjusting for age, stage and grade in samples from the COGS dataset.**

## Abbreviations

BCSS: breast cancer-specific survival
CI: confidence interval
COGS: Collaborative Oncological Gene-environment Study
ER: oestrogen receptor
GWAS: genome-wide association study (studies)
HER2: human epidermal growth factor receptor 2
HR: hazard ratio
SNP: single nucleotide polymorphism
