# Supplementary material for: Common germline polymorphisms associated with breast cancer-specific survival
Source: Breast Cancer Res. 2015 Apr 22;17(1):58. doi: 10.1186/s13058-015-0570-7 (PMC4484708; doi:10.1186/s13058-015-0570-7)
Supplement: Additional file 1: — Study information for GWAS included in meta-analysis [ 8 ]. [file 13058_2015_570_MOESM1_ESM.pdf]

Additional File 1 – Summary of the participating studies [Guo, Schmidt, Pharoah et al, under review]

Study	Genotyping platform	Age	Country	Description of the study
COGS	Illumina iSelect	16-96	International	Collaborative Oncological Gene-Environment Study
CGEMS	Illumina Hap550K	44-83	USA	NHS cases from nurses’ health study genotypes as part of CGEM project
SASBAC	Illumina HumanHap300 and HumanHap240S	50-75	Sweden	Cases from Swedish Case-control study, part of BCAC
UK2	Illumina 670k	17-88	UK	Consist of National study of breast cancer of age<41, and Subset of samples from national familial breast cancer study
Metabric	Affymetrix SNP 6.0	26-96	UK	UK samples from international breast cancer genomics project
PG-SNPs	Affymetrix SNP 6.0	22-77	UK	UK samples from breast cancer chemotherapy treatment response study
HEBCS	Illumina 550K	26-87	Finland	Helsinki Breast Cancer Study
BPC3-CPSII	Illumina 660W	51-89	USA	The National Cancer Institute Breast and Prostate Cancer Cohort Consortium: American Cancer Society Cancer Prevention Study-II
BPC3-EPIC	Illumina 660W	27-75	Europe	The National Cancer Institute Breast and Prostate Cancer Cohort Consortium: European Prospective Investigation of Cancer
BPC3-NHS2	Illumina 550K	44-83	USA	The National Cancer Institute Breast and Prostate Cancer Cohort Consortium: Nurses' Health Studies II
