# Supplementary material for: Common germline polymorphisms associated with breast cancer-specific survival
Source: Breast Cancer Res. 2015 Apr 22;17(1):58. doi: 10.1186/s13058-015-0570-7 (PMC4484708; doi:10.1186/s13058-015-0570-7)
Supplement: Additional file 2 — Samples included in meta-analysis by study [ 8 ]. [file 13058_2015_570_MOESM2_ESM.pdf]

Additional File 2 – Samples included in meta-analysis by study [Guo, Schmidt, Pharoah et al, under review]

	All Cases		ER positive		ER negative	
**Study**	**N (breast cancer deaths)**	**Person-years**	**N (breast cancer deaths)**	**Person-years**	**N (breast cancer deaths)**	**Person-years**
COGS	29 360 (1790)	128 552	20 605 (942)	89 702	4926 (558)	21 437
CGEMS	1145 (93)	7711	--	--	--	--
SASBAC	787 (69)	3739	483 (44)	2294	108 (9)	502
UK2	2763 (233)	23 112	--	--		
Metabric	369 (86)	1570	291 (59)	1268	63 (25)	225
PG-SNPs	1786 (204)	5820	1188 (116)	3916	586 (87)	1888
HEBCS	742 (285)	4666	492 (172)	3458	196 (101)	982
BPC3-CPSII	293 (30)	2544	--	--	293 (30)	2544
BPC3-EPIC	476 (74)	2226	--	--	476 (74)	2226
BPC3-NHS2	233 (36)	2732	--	--	233 (36)	2732
**Total**	**37 954 (2900)**		**23 059 (1333)**		**6881 (920)**	
