# Supplementary material for: Common germline polymorphisms associated with breast cancer-specific survival
Source: Breast Cancer Res. 2015 Apr 22;17(1):58. doi: 10.1186/s13058-015-0570-7 (PMC4484708; doi:10.1186/s13058-015-0570-7)
Supplement: Additional file 3: — Previously reported associations with breast cancer survival. [file 13058_2015_570_MOESM3_ESM.pdf]

Additional File 3 - Previously reported associations with breast cancer survival

SNP	Gene	Author	Study	Main Population Ethnicity	Number of Cases	Genotype	OS HR	DFS HR	BCSS HR	Comments
rs9344	CCND1	Bewick et al (2006)	Study conducted by Sudbury Regional Hospital, Sudbury, Ontario, Canada	CEU	95 patients with MBC.	AA<br>AG<br>GG<br>P-value	NR	1.0<br>0.8(0.5-1.33)<br>1.6(0.9-2.69)<br>0.03	1.0<br>0.8(0.48-1.35)<br>1.5(1.89-2.67)<br>0.03	HRs adjusted for age
rs2479717	CCND3	Azzato et al (2008)	SEARCH (Studies of Epidemiology and Risk factors in Cancer Heredity). Includes female breast cancer patients in the region ECRIC.	CEU	4470	AA<br>AT<br>TT<br>P-value	1.0<br>1.27(1.08-1.5)<br>1.43(1.08-1.91)<br>0.0001	NR	1.0<br>1.25(1.04-1.5)<br>1.46(1.08-1.99)<br>0.001	
rs52812045	CD24	Buck et al (2013)	Mammographic Risk Factor Investigation study (MARIE)	CEU	2514	CC & CT<br>TT<br>P-value	1.0<br>1.52 (1-2.3)<br>0.05	NR	1.0<br>1.83 (1.1-3.05)<br>0.02	HRs adjusted for tumour size,nodes,grade,ER/PR, HER2,diabetes,detection, HRT,radiotherapy, age.
rs13347	CD44	Jiang et al (2012)	Patients from First Affiliate Hospital of Soochow University and tumor hospitals affiliated with Guangzhou Medical College.	CHB	897	CC<br>CT<br>TT<br>P-value	1.0<br>1.54 (1.02-2.3)<br>2.84 (1.80-4.4)<br>< 0.0001	NR	NR	Adjusted for age, BMI, family history, TNM stage, estrogen receptor status, progesterone receptor status, pathological type, menopausal status and age at menarche.<br>Pooled discovery and validation.
rs4860	COMT	Long et al (2007)	Shanghai Breast Cancer Study (SBCS)	CHB	1140	GG<br>AA	NR	1.0<br>1.6(1.1-2.4)	NR	
rs6504950	COX11	Bayraktar et al (2013)	Early-Stage Breast Cancer Repository (ESBCR)	CEU	739	GG/AG<br>AA<br>P-value	1.0<br>1.7 (1.1-2.6)<br>0.004	1.0<br>1.2(0.7-2.0)<br>0.42	NR	
rs4646903	CYP1A1	Long et al (2007)	As above	CHB	1140	TT<br>CC	1.0<br>1.4(1.0-2.1)	1.0<br>1.6 (1.2-2.2)	NR	
rs10046	CYP19A1	Fasching et al (2008)	The Bavarian Breast Cancer and Controls (BBCC) study approved by the Ethics Committee of the Friedrich-Alexander-University Erlangen-Nuremberg.	CEU	1257	TT/TC<br>CC<br>P-value	NR	1.0<br>0.51(0.32-0.81)<br>0.004	NR	HRs adjusted for age, nodal status, tumour size, grading, and hormone receptor status

SNP	Gene	Author	Study	Main Population Ethnicity	Number of Cases	Genotype	OS HR	DFS HR	BCSS HR	Comments
rs12900137	CYP19A1	Long et al (2006)	Shanghai Breast Cancer Study (SBCS)	CHB	1136	GG/CG<br>CC<br>P-value	1.0<br>1.7 (0.9-3.2)<br>0.1	1.0<br>2.1 (1.2-3.6)<br>0.01	NR	HRs adjusted for age
rs1902586	CYP19A1	Long et al (2006)	As above	CHB	1136	GG/AG<br>AA<br>P-value	1.0<br>1.2 (0.8-1.8)<br>0.38	1.0<br>1.4 (1.0-2.0)<br>0.04	NR	HRs adjusted for age
rs700519	CYP19A1	Long et al (2006)	As above	CHB	1136	CC/CT<br>TT<br>P-value	1.0<br>2.2 (1.2-4.1)<br>0.02	1.0<br>2.1 (1.1-3.9)<br>0.02	NR	HRs adjusted for age
rs730154	CYP19A1	Long et al (2006)	As above	CHB	1136	TT/CT<br>CC<br>P-value	1.0<br>1.2 (0.8-1.8)<br>0.34	1.0<br>1.5 (1.1-2.0)<br>0.02	NR	HRs adjusted for age
rs936306	CYP19A1	Long et al (2006)	As above	CHB	1136	CC/CT<br>TT<br>P-value	1.0<br>1.2 (0.8-1.7)<br>0.42	1.0<br>1.4 (1.0-2.0)<br>0.04	NR	HRs adjusted for age
rs28566535	CYP19A1	Long et al (2006)	As above	CHB	1136	AA/AC<br>CC<br>P-value	1.0<br>1.2 (0.8-1.7)<br>0.51	1.0<br>1.5 (1.1-2.1)<br>0.02	NR	HRs adjusted for age
rs2070744	ENOS (NOS3)	Choi et al (2006)	Population-based case–control study in Seoul, Korea	CHB	873	TT<br>CT/CC	NR	1.0<br>2.1(1.03–4.33)	NR	HR adjusted for ER/PR status,<br>lymph node involvement, and tumour size
rs13181	ERCC2	Bewick et al (2011)	Patients from Sudbury Regional Hospital, Sudbury, Ontario	CEU	95	AA/AC<br>CC<br>P-value	NR	NR	1.0<br>1.9 (1.06–3.26)<br>0.04	HR adjusted for age
rs2981582	FGFR2	Bayraktar et al (2013)	Early-Stage Breast Cancer Repository (ESBCR)	CEU	739	GG/AG<br>AA<br>P-value	1.0<br>0.6 (0.4-0.9)<br>0.04	1.0<br>1.0 (0.7-1.4)<br>0.382	NR	
rs7121	GNAS1	Otterbach et al (2006)	Study from Institute of Pathology<br>and Neuropathology (University Hospital of Essen,<br>Germany).	CEU	279	CC<br>CT<br>TT<br>P-value	1.0<br>1.72(0.91–3.25)<br>2.08(1.01–4.27)<br>0.045	NR	NR	HR adjusted for gender,<br>pT-stages, grade,<br>age, oestrogen receptor status.
rs1695	GSTP1	Sweeney et al (2000)	Patients at the Arkansas Cancer Research Center, US.	CEU	240	AA<br>AG<br>GG<br>P-value	1.0<br>0.8 (0.5–1.3)<br>0.3 (0.1–1.0)<br>0.05	1.0<br>1.0(0.6 –1.6)<br>0.7(0.3–1.8)<br>> 0.05	NR	HRs adjusted for age, race, stage at diagnosis, node status, and ER and PR status.

SNP	Gene	Author	Study	Main Population Ethnicity	Number of Cases	Genotype	OS HR	DFS HR	BCSS HR	Comments
		Duggan et al (2013)	Health, Eating,Activity and Lifestyle (HEAL)	Mixed	533	AA<br>AG<br>GG<br>P-value	1.0<br>1.98 (1.3-3.1)<br>1.24 (0.6-2.6)<br>0.08	NR	1.0<br>1.7(0.9-3.2)<br>0.6(0.2-2.3)<br>0.27	HR adjusted for race, ethnicity,study site, bmi,stage and treatment
rs1800795	IL6	DeMichele et al (2003)	Node positive, high-risk patients at University of Pennsylvania Cancer Center	CEU	124	GC/CC<br>GG<br>P-value	1.0<br>2.6 (1.2–5.8)<br>0.02	1.0<br>2.1 (1.1–4.1)<br>0.035	NR	HRs adjusted for ER status, lymph node group, and tumor size
rs1800872	IL10	Gerger et al (2010)	Study conducted at Division of Oncology, Department of Internal Medicine, Medical University of Graz, Austria	CEU	432	CC<br>CA/AA<br>P-value	NR	1.0<br>1.48(1.07-2.04)<br>0.019	NR	HRs adjusting for age at diagnosis, tumor size, lymph node status, clinical stage, histological grade, ER, PR and treatment modalities
rs12508721	IL21	You et al (2013)	First Affiliate Hospital of Soochow University (N=566) and Tumor Hospitals affiliated with Guangzhou Medical Uni(N=325). Han Chinese population.	CHB	891	CC<br>CT<br>TT<br>P-value	1.0<br>0.48 (0.31–0.74)<br>0.38(0.19-0.74)<br><0.0001	NR	NR	HRs adjusted for age, age at menarche (yrs), menstrual status, BMI, pathological type, stage, ER/PR status, and family history of any cancer
rs1805386	LIG4	Goode et al (2002)	ABC study - Anglian Breast Cancer. Population based study	CEU	325	TT/TC<br>CC	1.0<br>4.15 (1.85-9.30)	NR	NR	HRs adjusted for grade, stage, and tumor type.
rs9934948	LOC1005 06172	Shu et al (2012)	Shanghai Breast Cancer Study (SBCS) and Shanghai Breast Cancer Survival Study (SBCSS), Nurses' Health Study(NHS) (CGEMS)	CHB; CEU	6110; 1145	TT<br>TC<br>CC<br>P-value	1.0<br>1.31 (1.08–1.60)<br>1.67 (1.34–2.09)<br>5.75x10-6	NR	NR	
rs1477017	MMP2	Slattery et al (2013)	The Breast Cancer Health Disparities Study	Hispanic, MEX and CEU	3592	AA<br>AG/GG<br>P-value	1.0<br>0.64 (0.49-0.85)<br>0.002	NR	NR	HRs adjusted for age. study site, BMI,stage

SNP	Gene	Author	Study	Main Population Ethnicity	Number of Cases	Genotype	OS HR	DFS HR	BCSS HR	Comments
rs1992116						CC<br>CT/TT<br>P-value	1.0<br>0.68 (0.52-0.89)<br>0.006	NR	NR	HRs adjusted for age. study site, BMI,stage
rs243836						GG<br>GA<br>AA<br>P-value	1.0<br>1.15 (0.83-1.59)<br>1.6 (1.1–2.31)<br>0.013	NR	NR	HRs adjusted for age. study site, BMI,stage
rs11639960						AA<br>AG/GG<br>P-value	1.0<br>0.59 (0.45-0.78)<br><0.001	NR	NR	HRs adjusted for age. study site, BMI,stage
rs11225297	MMP7	Beeghly-Fadiel et al (2009)	Shanghai Breast Cancer Study (SBCS)	CHB	1079	AA<br>AT<br>TT<br>P-value	1.0<br>0.7(0.5–0.9)<br>0.3(0.1-0.8)<br>0.001	1.0<br>0.8 (0.6–1.1)<br>0.4 (0.2–0.8)<br>0.007	NR	HRs adjusted for age, disease stage, ER, PR, menopausal status, treatment
		Beeghly-Fadiel et al (2012)	As above	CHB	5192	AA<br>AT<br>TT<br>P-value	1.0<br>0.8 (0.7–1.0)<br>0.4 (0.2–0.8)<br>0.003	1.0<br>0.9 (0.8–1.1)<br>0.8 (0.5–1.2)<br>0.095	NR	As above
rs11225395	MMP8	Beeghly-Fadiel et al (2012)	As above	CHB	5192	CC<br>CT<br>TT<br>P-value	1.0<br>0.9 (0.7–1.1)<br>0.6 (0.4–0.9)<br>0.021	1.0<br>0.9 (0.7–1.1)<br>0.9 (0.7–1.2)<br>0.353	NR	As above
	(TNM 0-2)	Decock et al (2007)	The Leuven Breast Cancer Study. The Shanghai Breast Cancer Study.	CHB	140	CC<br>CT/TT<br>P-value	1.0<br>0.7 (0.5 -1.0)<br>0.02	1.0<br>0.7 (0.5 - 0.9)<br>0.02	NR	TNM stage 0-2 patients
rs2333227	MPO	Ambrosone et al (2005)	Study approved by the Institutional Review Board of the University of Arkansas for Medical Sciences. Study conducted at Arkansas Cancer Research Center.	CEU; YRI	279	AA/GA<br>GG<br>P-value	1.0<br>0.60 (0.38-0.95)<br>0.03	1.0<br>0.52(0.29-0.92)<br>0.02	NR	HRs adjusted for age, stage with node status at diagnosis, race, ER status, and PR status

SNP	Gene	Author	Study	Main Population Ethnicity	Number of Cases	Genotype	OS HR	DFS HR	BCSS HR	Comments
rs1801131	MTHFR	Martin et al (2006)	Patients recruited at University of Maryland Medical Center, the Baltimore Veterans Affairs Medical Center, Union Memorial Hospital, Mercy Medical Center, and the Sinai Hospital in Baltimore, Maryland.	CEU; YRI	198	AA<br>AC/CC<br>P-value	NR	NR	1.0<br>2.05(1.05-4.00)<br>0.03	HRs adjusted for age at diagnosis, race, C677T, BMI, estrogen receptor, TNM stage, and chemotherapy
rs16949649	NME1	Qu et al (2008)	Shanghai Breast Cancer Study (SBCS)	CHB	1134	TT<br>CT/CC	NR	1.0<br>1.3 (1.0-1.6)	1.0<br>1.4(1.1-1.9)	HRs adjusted by age, education, TNM stage, radiotherapy, chemotherapy, tamoxifen use, and ER/PR status
rs2302254	NME1	Qu et al (2008)	As above	CHB	1134	CC<br>CT/TT	NR	1.0<br>1.3 (1.0-1.6)	1.0<br>1.2 (0.9-1.5)	As above
rs1800566	NQO1	Fagerholm et al (2008)	Finnish studies- Helsinki region, Tampere and Kuopio regions	CEU	1005; 1162	TC/CC<br>TT	1.0/1.0<br>3.08(1.66–5.71)/<br>2.00 (1.23–3.27)	1.0/1.0<br>1.76(0.9-3.45)/<br>0.32(0.04–2.28)	NR	Relative Risks.<br>Discovery/Validation
rs2886162	NFE2L2 (NRF2)	Hartikainen et al (2012)	Kuopio Breast Cancer Project (KBCP)	CEU	219	GG/GA<br>AA<br>P-value	1.0<br>1.69(1.05–2.72)<br>0.032	NR	NR	HRs adjusted for tumor grade, nodal status, ER/PR status, histologic type, tumor size, HER2 status, cytoplasmic NRF2 expression
rs4778137	OCA2	Azzato et al (2010)	SEARCH (Studies of Epidemiology and Risk factors in Cancer Heredity). Includes female breast cancer patients in the region ECRIC.	CEU	17 467<br><br>3154	Per G allele<br>P-value<br><br>P-value	0.93 (0.87-0.98)<br>0.011<br><br>0.82 (0.73-0.92)<br>5x10-4	NR	NR	All patients<br><br>ER negative patients<br><br>GWAS.

SNP	Gene	Author	Study	Main Population Ethnicity	Number of Cases	Genotype	OS HR	DFS HR	BCSS HR	Comments
rs1799889	SERPINE 1 (PAI-1)	Zhang et al (2006)	Shanghai Breast Cancer Study-Chinese population based case–control study.	CHB	1083	5G/5G<br>5G/4G<br>4G/4G<br>P-value	1.0<br>1.2 (0.8-1.7)<br>1.5 (1.0-2.3)<br>0.07	1.0<br>1.2 (0.8-1.7)<br>1.7 (1.1-2.4)<br>0.007	NR	HRs adjusted for age, education, body mass index, disease stage, ER and PR status, chemotherapy, radiotherapy, surgery, and tamoxifen use.
rs4251864	PLAUR	Pande et al (2013)	Early Stage Breast Cancer Repository cohort at MD Anderson Cancer Center	CEU	1029	TT<br>TC<br>CC<br>Per-allele<br>P-value	NR	1.0<br>1.14(1.07-1.87)<br>1.95(1.06-3.59)<br>1.4(1.13-1.75)<br>$2.2x10^{-3}$	NR	
rs10477313	PPP2R2B	Jamshidi et al (2013)	Four European studies within the BCAC	CEU	4701	GG<br>GA/AA<br>P-value	1.0<br>0.83(0.7-0.9)<br>0.034	NR	NR	HRs adjusted for grade, tumor size, nodal status, primary metastasis, ER/PR, study and age of diagnosis.<br>Association due to hormonal treatment
rs1029946	PRKAG2	As above	As above	CEU		AA/AG<br>GG<br>P-value	1.0<br>0.57(0.3-0.9)<br>0.044	NR	NR	As above + TP53 status
rs3784099	RAD51B	Shu et al (2012)	Shanghai Breast Cancer Study (SBCS) and Shanghai<br>Breast Cancer Survival Study (SBCSS), Nurses' Health Study (NHS)	CHB; CEU	5245	GG<br>AG<br>AA<br>P-value	1.0<br>1.41 (1.18–1.68)<br>2.64 (1.74–4.03)<br>1.17x10-7	NR	NR	GWAS. Not replicated in European population.
rs881658	RXRA	Pande et al (2013)	Early Stage Breast Cancer Repository cohort at MD Anderson Cancer Center	CEU	1029	GG<br>GA<br>AA<br>Per-allele<br>P-value	NR	1.0<br>1.33(1.02-1.73)<br>1.93(1.36-2.72)<br>1.38(1.16-1.63)<br>$2.1x10^{-4}$	NR	

SNP	Gene	Author	Study	Main Population Ethnicity	Number of Cases	Genotype	OS HR	DFS HR	BCSS HR	Comments
rs11185659						CC<br>CT<br>TT<br>Per-allele<br>P-value	NR	1.0<br>1.42(1.1-1.82)<br>2.0(1.25-3.19)<br>1.41(1.17-1.71)<br>$3.8x10^{-4}$	NR	
rs10881583						TT<br>TC<br>CC<br>Per-allele<br>P-value	NR	1.0<br>1.21(0.92-1.58)<br>1.81(1.30-2.51)<br>1.32(1.12-1.56)<br>$7.7x10^{-4}$	NR	
rs881657						TT<br>TC<br>CC<br>Per-allele<br>P-value	NR	1.0<br>1.26(0.97-1.64)<br>1.77(1.24-2.52)<br>1.32(1.11-1.56)<br>$1.8x10^{-3}$	NR	
rs7864987						TT<br>TC<br>CC<br>Per-allele<br>P-value	NR	1.0<br>1.19(0.91-1.54)<br>1.75(1.21.2.51)<br>1.28(1.08-1.53)<br>$4.6x10^{-3}$	NR	
rs5361	SELE	Kontogianni et al (2013)	Study from patients at First Propaedeutic Department of Surgery of the ‘‘Hippocration’’ University Hospital of Athens, Greece	CEU	261	CC<br>AC<br>AA	Reference<br>0.002<br>0.008	NR	NR	P-values reported. CC genotype associated with lower overall survival.
rs3741378	SIPA1	Pei et al (2013)	Study at Breast Center, Peking University Cancer Hospital.	CHB	185	CC/CT<br>TT<br>P-value	1.0<br>2.16(1.12–4.15)<br>0.022	NR	NR	HRs adjusted for age, menopausal status, HER2 status, ER or PR status
rs7867504	SLC28A3	Lee et al (2013)	MBC patients from a PG study at Samsung Medical Centre and Seoul Nat. Uni. Hospital	CHB	85	CC/CT<br>TT<br>P-value	1.0<br>2.6 (1.1-6.3)<br>0.027	NR	NR	

SNP	Gene	Author	Study	Main Population Ethnicity	Number of Cases	Genotype	OS HR	DFS HR	BCSS HR	Comments
rs3775775	SULT1E1	Choi et al (2005)	Population-based case–control study in Seoul, Korea	CHB	989	TT<br>TC/CC	NR	1.0<br>3.2 (1.39-7.48)	NR	HR adjusted for age and tumor-node-metastasis stage
rs1800470	TGF	Shu et al (2004)	As above	CHB	1111	TT<br>TC<br>CC	1.0<br>1.2 (0.8–1.8)<br>1.1 (0.7–1.6)	1.0<br>1.4 (1.0–2.1)<br>1.3 (0.9–1.9)	NR	HRs adjusted for age, education, TNM, radiotherapy, chemotherapy, ER&PR status and tamoxifen use.
rs8136803	TIMP3	Peterson et al (2009)	Shanghai Breast Cancer Study	CHB	1062	GG<br>GT<br>TT	1.0<br>1.0 (0.7-1.6)<br>1.9 (0.6-6.1)	1.0<br>1.1 (0.8-1.6)<br>3.9 (1.4-10.6)	NR	HRs adjusted for age, stage of disease, menopausal status, ER/PR, and treatment (chemotherapy, radiotherapy, and tamoxifen
rs11536889	TLR4	Yang et al (2013)	Patients from Lianyungang No.1 Hospital	CHB	604	GG<br>C allele<br>P-value	1.0<br>1.38(1.09–3.12)<br>0.017	NR	NR	
rs3803662	TOX3	Fasching et al (2012)	23 BCAC studies (OS) 16 BCAC studies (BCSS)	CEU	25 853	CC<br>CT<br>TT<br>P-value	1.0<br>0.97 (0.91–1.04)<br>1.21 (1.09–1.35)<br>0.0002	NR	1.0<br>1.01(0.92-1.11)<br>1.29(1.12-1.47)<br>0.0009	HRs adjusted for age at diagnosis, tumor size, nodal status and grade
rs1042522	TP53	Toyama et al (2007)	Study conducted at Nagoya City University Hospital, Nagoya, Japan	JPT	557	GG &GC<br>CC<br>P-value	NR	1.0<br>1.67(1.01-2.76)<br>0.047	NR	Relative Risks
		Tommiska et al (2005)	Study conducted by Helsinki University Central Hospital Finland.	CEU	888	GG&GC<br>CC<br>P-value	1.0<br>2.1 (1.4-3.3)<br>0.001	NR	NR	Relative Risks
	(p53 -ve tumours)	Schmidt et al (2009)	Pooled data from four breast cancer cohorts within the Breast Cancer Association Consortium	CEU	3749	GG&GC<br>CC<br>P-value	NR	NR	1.0<br>1.79(1.05-3.05)<br>0.03	HR adjusted for study, age, stage, grade and ER
rs731236	VDR	Perna et al (2013)	ESTHER II study and the VERDI study, Saarland, Germany	CEU	498	TT<br>TC<br>CC	1.0<br>1.5(0.7-3.1)<br>2.2(0.9-5.3)	NR	1.0<br>2.0(0.9-4.9)<br>3.0(1.1-8.1)	HR adjusted for age, stage,family history of breast cancer, mastectomy, and BMI

SNP	Gene	Author	Study	Main Population Ethnicity	Number of Cases	Genotype	OS HR	DFS HR	BCSS HR	Comments
rs2010963	VEGF	Lu et al (2005)	Shanghai Breast Cancer Study (SBCS)	CHB	1193	CC<br>GC<br>GG<br>P-value	1.0<br>1.2 (0.8-1.8)<br>1.6 (1.0-2.5)<br>0.02	1.0<br>0.7 (0.5-1.1)<br>1.0 (0.7-1.5)<br>0.48	NR	HR adjusted for age
rs25487	XRCC1	Bewick et al (2006)	Study conducted by Sudbury Regional Hospital, Sudbury, Ontario, Canada	CEU	95 patients with MBC.	GG<br>AG<br>AA<br>P-value	NR	1.0<br>1.0(0.77-1.95)<br>2.2(1.18-3.99)<br>0.04	1.0<br>0.9(0.58-1.44)<br>2.7(1.45-5.00)<br>0.001	
		Castro et al (2013)	EBC patients at Hospital Universitario de Salamanca between 1990 and 2004	CEU	150	AA<br>GG+AG<br>P-value	NR	1.0<br>2.5 (1.1-5.1)<br>0.035	NR	HR adjusted for tumour size
rs3218536	XRCC2	Lin et al (2011)	Sheffield Breast Cancer Study (SBCS), Utah Breast Cancer Study (UBCS), Breast Cancer Association Consortium (BCAC).	CEU	1131; 860; 8074	GG<br>GA<br>AA<br>P-value	1.0<br>1.19(0.76-1.89)<br>4.26(1.69-10.72)<br>4x10-6	NR	NR	HRs adjusted for age.
rs861539	XRCC3	Bewick et al (2006)	Study conducted by Sudbury Regional Hospital, Sudbury, Ontario, Canada	CEU	95 patients with MBC.	CC<br>CT<br>TT<br>P-value	NR	1.0<br>1.0(0.65 -1.61)<br>2.0(1.05-3.82)<br>0.06	1.0<br>0.9(0.62-1.54)<br>2.0(1.06-3.82)<br>0.05	
		Castro et al (2013)	EBC patients at Hospital Universitario de Salamanca between 1990 and 2004	CEU	150	CC<br>CT+TT<br>P-value	NR	1.0<br>2.8 (1.0-9.2)<br>0.049	NR	
rs251864	ZFP36	Upadhyay et al (2012)	Caucasian patients from University of South Carolina Cancer Research Center Biorepository in collaboration with Palmetto Health Tissue Bank, Columbia, SC	CEU	170	AA<br>AG<br>GG<br>P-value	1.0<br>2.19 (1.15–4.14)<br>1.50 (0.58–3.92)<br>0.0477	NR	NR	

NR – not reported, OS – overall survival, DFS – disease-free survival, BCSS – breast cancer specific survival
