# Supplementary material for: Common germline polymorphisms associated with breast cancer-specific survival
Source: Breast Cancer Res. 2015 Apr 22;17(1):58. doi: 10.1186/s13058-015-0570-7 (PMC4484708; doi:10.1186/s13058-015-0570-7)
Supplement: Additional file 4: — Look-up of previously reported associations in meta-analysis. [file 13058_2015_570_MOESM4_ESM.pdf]

Additional File 4 – Look up of previously reported associations in meta-analysis.

SNP	Gene	Published	Model	Effect Allele	Effect Allele Freq	HR (90% CI)	One-sided p-value
rs2981582	*FGFR2*	Bayraktar et al (2013)	Dominance	G	0.57	1.09 (**1.04** – 1.14)	**0.00085**
rs1800566	*NQO1*	Fagerholm et al (2008)	Dominance	A	0.19	1.10 (**1.03** -1.17)	**0.0046**
rs9934948	*LOC100506172*	Shu et al (2012) (GWAS)	Co-dominance	T	0.15	0.92 (0.86 - **0.98**)	**0.011**
rs1800470	*TGF*	Shu et al (2004)	Co-dominance	A	0.61	0.95 (0.91 - **0.99**)	**0.030**
rs3775775	*SULTIE1*	Choi et al (2005)	Dominance	G	0.09	1.08 (**1.00** -1.16)	**0.046**
rs731236	*VDR*	Perna et al (2013)	Co-dominance	G	0.39	1.04 (**1.00** – 1.08)	0.056
rs10477313	*PPP2R2B*	Jamshidi et al (2013)	Dominance	T	0.12	0.94 (0.87 - **1.01**)	0.081
rs700519	*CYP19A1*	Long et al (2006)	Dominance	A	0.03	1.10 (**0.98** – 1.22)	0.093
rs16949649	*NME1*	Qu et al (2008)	Dominance	C	0.39	1.03 (**0.99** – 1.07)	0.11
rs4251864	*PLAUR*	Pande et al (2013)	Co-dominance	G	0.10	1.04 (**0.96** – 1.12)	0.20
rs2333227	*MPO*	Ambrosone et al (2005)	Dominance	T	0.21	1.03 (**0.97** – 1.09)	0.20
rs8136803	*TIMP3*	Peterson et al (2009)	Co-dominance	T	0.07	1.03 (**0.95** – 1.11)	0.25
rs1805386	*LIG4*	Goode et al (2002)	Dominance	G	0.18	1.02 (**0.97** – 1.07)	0.25
rs1800795	*IL6*	DeMichele et al (2003)	Dominance	G	0.56	1.02 (**0.97** – 1.07)	0.26
rs25487	*XRCC1*	Bewick et al (2006), Castro et al (2013)	Co-dominance, dominance	C	0.65	0.98 (0.93 - **1.03**)	0.27
rs2010963	*VEGF*	Lu et al (2005)	Co-dominance	G	0.69	1.02 (**0.97** – 1.07)	0.27
rs2302254	*NME1*	Qu et al (2008)	Dominance	T	0.19	1.02 (**0.96** – 1.08)	0.27
rs11536889	*TLR4*	Yang et al (2013)	Co-dominance	C	0.14	1.03 (**0.95** – 1.11)	0.28
rs11225395	*MMP8*	Decock et al (2007), Beeghly-Fadiel et al (2012)	Dominance, co-dominance	G	0.56	1.02 (**0.97** – 1.07)	0.29
rs1042522	*TP53*	Schmidt et al (2009), Toyama et al (2007), Tommiska et al (2005)	Dominance	C	0.74	1.02 (**0.95** – 1.09)	0.31
rs5361	*SELE*	Kontogianni et al (2013)	Dominance	G	0.10	0.98 (0.91 - **1.05**)	0.32
rs2479717	*CCND3*	Azzato et al (2008)	Co-dominance	A	0.74	0.98 (0.92 - **1.04**)	0.32

Hazard ratios, confidence intervals and p-values are from a co-dominant model.

P-values refer to a one-sided test of association in the direction indicated in blue in the 90% CI of the HR.

Additional File 4 – Continued.

SNP	Gene	Published	Model	Effect Allele	Effect Allele Freq	HR (90% CI)	One-sided p-value
rs13181	*ERCC2*	Bewick et al (2011)	Dominance	G	0.37	1.01 (**0.97** – 1.05)	0.35
rs11225297	*MMP7*	Beeghly-Fadiel et al (2009,2012)	Co-dominance	T	0.12	0.98 (0.91 - **1.05**)	0.35
rs13347	*CD44*	Jiang et al (2012)	Co-dominance	T	0.20	1.01 (**0.96** – 1.06)	0.35
rs3218536	*XRCC2*	Lin et al (2011)	Co-dominance	T	0.08	1.02 (**0.94** – 1.10)	0.36
rs6504950	*COX11*	Bayraktar et al (2013)	Dominance	A	0.27	1.01 (**0.96** – 1.06)	0.38
rs2364725	*NRF2*	Hartikainen et al (2012)	Dominance	T	0.51	1.01 (**0.96** – 1.06)	0.39
rs1902586	*CYP19A1*	Long et al (2006)	Dominance	A	0.05	1.01 (**0.91** – 1.11)	0.44
rs11185659	*RXRA*	Pande et al (2013)	Co-dominance	T	0.21	1.00 (**0.94** – 1.06)	0.47
rs12900137	*CYP19A1*	Long et al (2006)	Dominance	C	0.05	1.00 (**0.91** – 1.09)	0.47
rs28566535	*CYP19A1*	Long et al (2006)	Dominance	C	0.05	1.00 (**0.90** – 1.10)	0.51
rs1029946	*PRKAG2*	Jamshidi et al (2013)	Dominance	G	0.12	1.00 (**0.93**- 1.07)	0.52
rs7867504	*SLC28A3*	Lee et al (2013)	Dominance	C	0.30	1.00 (**0.95** – 1.05)	0.53
rs881658	*RXRA*	Pande et al (2013)	Co-dominance	T	0.30	1.00 (**0.94** – 1.06)	0.54
rs881657	*RXRA*	Pande et al (2013)	Co-dominance	G	0.29	0.99 (**0.94** – 1.04)	0.56
rs1695	*GSTP1*	Sweeney et al (2000), Duggan et al (2013)	Co-dominance	G	0.33	1.00 (**0.95** – 1.05)	0.57
rs1801131	*MTHFR*	Martin et al (2006)	Dominance	G	0.32	0.99 (**0.94** – 1.04)	0.62
rs7864987	*RXRA*	Pande et al (2013)	Co-dominance	C	0.25	0.99 (**0.93** – 1.05)	0.64
rs10881583	*RXRA*	Pande et al (2013)	Co-dominance	C	0.25	1.02 (0.96 - **1.08**)	0.66
rs2070744	*eNOS*	Choi et al (2006)	Dominance	T	0.62	1.01 (0.96 - **1.06**)	0.67
rs730154	*CYP19A1*	Long et al (2006)	Dominance	C	0.15	0.98 (**0.92** – 1.04)	0.67
rs936306	*CYP19A1*	Long et al (2006)	Dominance	T	0.15	0.98 (**0.92** – 1.04)	0.70
rs1800872	*IL10*	Gerger et al (2010)	Dominance	G	0.77	1.02 (0.97 - **1.07**)	0.72
rs3741378	*SIPA1*	Pei et al (2013)	Dominance	T	0.13	0.97 (**0.91** – 1.03)	0.76
rs3784099	*RAD51B*	Shu et al (2012) (GWAS)	Co-dominance	A	0.29	0.98 (**0.93** – 1.03)	0.77

Hazard ratios, confidence intervals and p-values are from a co-dominant model.

P-values refer to a one-sided test of association in the direction indicated in blue in the 90% CI of the HR.

Additional File 4 – Continued.

SNP	Gene	Published	Model	Effect Allele	Effect Allele Freq	HR (90% CI)	One-sided p-value
rs861539	*XRCC3*	Bewick et al (2006), Castro et al (2013)	Co-dominance, dominance	A	0.37	0.98 (**0.93** – 1.03)	0.79
rs11639960	*MMP2*	Slattery et al (2013)	Dominance	G	0.35	1.03 (0.98- **1.08**)	0.86
rs10046	*CYP19A1*	Fasching et al (2008)	Dominance	A	0.54	0.97 (**0.93** – 1.01)	0.86
rs7121	*GNAS1*	Otterbach et al (2006)	Co-dominance	T	0.47	0.96 (**0.91** – 1.01)	0.88
rs251864	*ZFP36*	Upadhyay et al (2012)	Co-dominance	G	0.31	0.95 (**0.90** – 1.00)	0.93
rs243836	*MMP2*	Slattery et al (2013)	Co-dominance	A	0.48	0.95 (**0.90** – 1.00)	0.98
rs12508721	*IL21*	You et al (2013)	Co-dominance	T	0.26	1.07 (1.02 - **1.12**)	0.98
rs1992116	*MMP2*	Slattery et al (2013)	Dominance	A	0.44	1.06 (1.01 - **1.11**)	0.99
rs4646903	*CYP1A1*	Long et al (2007)	Dominance	G	0.10	0.89 (**0.83** – 0.95)	0.99
rs1477017	*MMP2*	Slattery et al (2013)	Dominance	G	0.36	1.07 (1.02 - **1.12**)	0.99

Hazard ratios, confidence intervals and p-values are from a co-dominant model.

P-values refer to a one-sided test of association in the direction indicated in blue in the 90% CI of the HR.
