# Supplementary material for: Common germline polymorphisms associated with breast cancer-specific survival
Source: Breast Cancer Res. 2015 Apr 22;17(1):58. doi: 10.1186/s13058-015-0570-7 (PMC4484708; doi:10.1186/s13058-015-0570-7)
Supplement: Additional file 5: — Multivariable analysis results adjusting for age, stage and grade in samples from the COGS dataset. [file 13058_2015_570_MOESM5_ESM.pdf]

Additional File 5 – Multivariable analysis results adjusting for age, stage and grade in samples from the COGS dataset.

		Unadjusted (N=37,954)		Adjusted (N=29,360 from COGS study)	
	**SNP**	**HR (90% CI)**	**One-sided P-value**	**HR (90% CI)**	**One-sided P-value**
**All**	rs2981582	1.09 (**1.04**-1.14)	**0.00085**	1.04 (**0.94**-1.15)	0.23
	rs1800566	1.10 (**1.03**-1.17)	**0.0046**	1.02 (**0.89**-1.16)	0.38
	rs9934948	0.92 (0.86-**0.98**)	**0.011**	1.02 (0.90-**1.17**)	0.65
	rs1800470	0.95 (0.91-**0.99**)	**0.030**	0.95 (0.86-**1.05**)	0.22
	rs3775775	1.08 (**1.00**-1.16)	**0.046**	1.17 (**1.01**-1.36)	**0.04**
**ER positive**	rs700519	1.30 (**1.10**-1.50)	**0.0050**	0.80 (**0.53**-1.20)	0.82
	rs731236	1.09 (**1.02**-1.16)	**0.017**	1.06 (**0.92**-1.21)	0.25
	rs12900137	1.18 (**1.02**-1.34)	**0.032**	1.08 (**0.79**-1.46)	0.35
	rs10477313	0.88 (0.77-**0.99**)	**0.035**	1.31 (1.03-**1.66**)	0.97
	rs2333227	1.09 (**1.01**-1.17)	**0.036**	1.22 (**1.04**-1.44)	**0.022**
	rs1902586	1.16 (**1.01**-1.31)	**0.041**	0.98 (**0.73**-1.33)	0.54
	rs28566535	1.15 (**1.00**-1.30)	**0.046**	1.04 (**0.78**-1.39)	0.42

Hazard ratios, confidence intervals and p-values are from a co-dominant model.
P-values refer to a one-sided test of association in the direction indicated in blue in the 90% CI of the HR.
Adjusted models were adjusted for age, stage and grade in 29,360 samples from the COGS dataset.
